# Case Report: PNPLA2 Gene Complex Heterozygous Mutation Leading to Neutral Lipid Storage Disease With Myopathy

**DOI:** 10.3389/fnint.2020.554724

**Published:** 2021-01-22

**Authors:** Jiejing Shi, Qianqian Qu, Haiyan Liu, Yan Zhang, Wenhao Cui, Ping Chen, Haidong Lv

**Affiliations:** ^1^Department of Neurology, Jiaozuo People’s Hospital, Jiaozuo, China; ^2^Jiaozuo People’s Hospital Affiliated to Xinxiang Medical College, Xinxiang, China

**Keywords:** neutral lipid storage disease with myopathy, PNPLA2 gene mutation, muscle imaging, muscle pathology, Jordans’ body

## Abstract

**Objective**: To investigate the clinical features, skeletal muscle imaging, muscle pathology, blood smear and so on of neutral lipid storage disease with myopathy (NLSDM) caused by PNPLA2 gene mutation.

**Methods**: The clinical data, skeletal muscle imaging, pathological data, and genetic test results of a patient with NLSDM treated in our hospital were collected in detail, and the previous literature was reviewed and compared.

**Results**: The main symptoms were muscle weakness and muscular atrophy. Pathological findings of muscle biopsy showed fat deposition in muscle fibers with border cavitation. Fatty droplets were seen in the cytoplasm of neutrophils in peripheral blood. Magnetic resonance imaging of the muscles of both lower extremities showed that muscle in the thigh *vastus intermedius*, lateral muscles, biceps, and the muscle abdominal area of the middle leg were filled or replaced by fat. Genetic test results suggested mutations in the PNPLA2 gene.

**Conclusion**: NLSDM is a rare clinical myopathy with abnormal lipid metabolism. Characteristic changes can be seen in skeletal muscle imaging and pathology. The detection of PNPLA2 gene mutation is an important basis for diagnosing NLSDM. Asymmetry and progressive limb weakness are the clinical features. Muscle MRI is mainly involved in the posterior group of the lower limbs. Jordans bodies in the peripheral blood smear and a large number of coarse-grained lipid deposits with rimmed vacuoles in muscle fibers are the characteristic pathological changes.

## Introduction

Neutral lipid storage disease (NLSD) is a group of rare autosomal recessive diseases characterized by a deficit in the degradation of neutral lipid, including neutral lipid storage disease with ichthyosis (NLSDI) or with myopathy (NLSDM; Missaglia et al., 2019). We report on a case of NLSD with myopathy confirmed by muscle pathology and genetic testing and review it in the literature.

## Materials and Methods

### Clinical Features

The patient was a 52-year-old female farmer. She visited our hospital on June 20, 2019. The main complaint was “progressive weakness of the limbs for 19 years.” At the age of 33, the patient gradually developed weakness in both lower limbs and was unable to run without any obvious inducement. She developed weakness of her right upper limb at the age of 36, and a year later her left upper limb was involved, unable to hold children, and she had difficulty standing up when climbing stairs and squatting but could do general housework. She went to the county hospital for treatment. Serum creatinine levels were significantly elevated in laboratory tests, and prednisone 60 mg/days was prescribed for the diagnosis of polymyositis. There was no improvement after 45 days of treatment, and then the dose was gradually reduced to zero. When the patient was 40 years old (2007), she came to our hospital for the first time. A muscle biopsy found that there were a lot of lipid deposits in the muscle fibers, accompanied by a large number of rimmed vacuoles. Treatment for lipid deposition myopathy showed no improvement. When she was 48 years old (2015), the limb weakness was further aggravated, her upper limbs could not be lifted over her shoulders, and she could not walk independently. She came to our hospital again. The results of a physical examination (the MRC-scale was used to grade muscle strength) were the following: neck extensor strength level 2, with obvious drooping head, proximal muscle strength level 3, and distal upper limbs level 2. The proximal muscle strength of both lower extremities is Grade 2, and the distal muscle strength is Grade 0. The proximal and distal muscles of the limbs were significantly atrophied, the muscle tension of the limbs was low, the tendon reflexes disappeared, the sensory system was normal, and the bilateral pathological signs were negative (−). There was no muscle tenderness or fasciculus fibrillation. Extremities and trunk skin were normal.

### Muscle Pathology

After the patient signed the informed consent form, an open biopsy was taken under local anesthesia, and the left gastrocnemius muscle was selected as the biopsy site. The muscle samples were fixed with liquid nitrogen; cut into frozen sections; stained by histochemistry, including HE, GT, ORO, PAS, NADH and ATPase staining; and observed under light microscope. In addition, peripheral blood smears were taken for conventional Wright staining and ORO staining, and the corpuscles in the neutrophils were observed under a high-powered oil microscope.

### Muscle MRI

Axial scanning of the patient’s lower limbs was performed using a GE 3.0T nuclear magnetic resonance machine. The scanning sequences included T1WI, and T2IDEAL.

### Genetic Testing

Genetic testing was performed on patients with informed consent. Genomic DNA was extracted from 4 ml venous blood and screened for genome-wide exon single-gene genetic diseases.

### Neuroelectrophysiology

Motor conduction velocity (MCV), sensory conduction velocity (SCV), and concentric circular needle electromyography (EMG) were measured using a Japanese photoelectric MEB-9200K myoelectric evoked potential meter.

## Results

### Muscle Pathology

**Figure 1:**
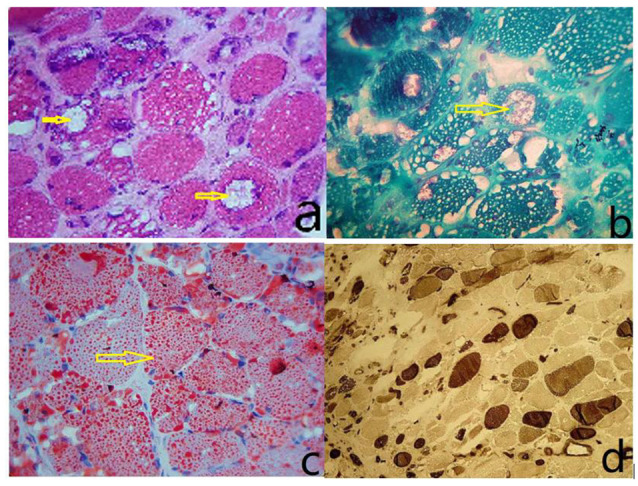
**(A)** The size of the muscle fibers was notably different, with small round or striated atrophic muscle fibers. Most of the muscle fibers had uniform small sieve orifice-like vacuoles, and marginal vacuoles of different sizes could be seen in some of the muscle fibers. Basophilic particle deposition was found in the vacuole edges and vacuoles (HE staining, ×400). **(B)** Improved Gomori tricolor showed that the edge of edging cavitation and particles deposited in the cavitation were dyed red, showing a typical edging cavitation (RV) but no broken red edge fiber (RRF; MGT staining, ×400). **(C)** ORO staining showed that a large amount of fat particles were deposited in most muscle fibers, and fat droplets were relatively thick (ORO staining, ×400). **(D)** Results showed I, II basic normal muscle fiber type distribution, without a group of network stations (ATPase10.6 staining, ×200).

The size of muscle fibers was obviously different; most of the muscle fibers had uniform small sieve orifice-like vacuoles, and marginal vacuoles of different sizes could be seen in some of the muscle fibers. Basophilic particle deposition was found in the vacuole edges and vacuoles. ORO staining showed that a large amount of fat particles were deposited in most muscle fibers, and the distribution of the two types of muscle fibers is roughly normal (Figure 1).

### Peripheral Blood Smear

**Figure 2:**
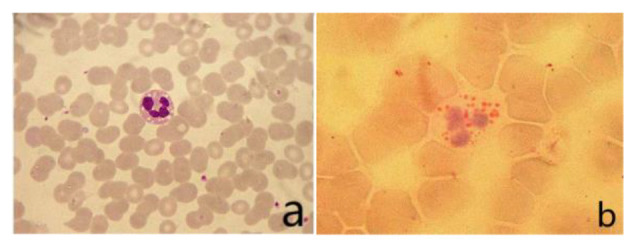
**(A)** Vacuoles could be seen in the cytoplasm of neutrophils in peripheral blood smear (Wright’s staining ×1,000). **(B)** Visible fat droplet deposition in the cytoplasm of neutrophils (Jordans’ bodies; “ORO” staining ×1,000).

The vacuoles in the cytoplasm of neutrophils can be seen in the peripheral blood smear. The vacuoles in the cytoplasm of neutrophils stained with “ORO” were stained as fat red droplets, showing a typical Jordans’ body (Figure 2).

### MRI Scan

**Figure 3:**
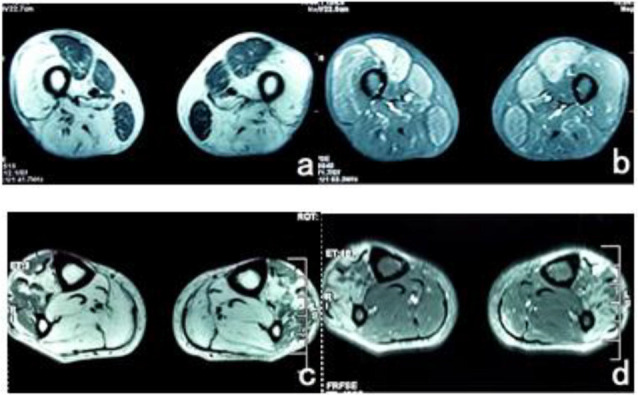
**(A)** T1WI sequence showed low signals in bilateral femoral *rectus femoris*, medial muscle, and *gracilis* muscle, and the remaining thigh muscles showed significantly high signals. **(B)** The muscle groups of the front and medial thighs of the T2 IDEAL showed slightly higher diffuse signals, indicating edema between the muscle tissues. **(C)** There were uneven low signals in the *tibialis anterior* muscles of both legs of T1WI, and diffuse high signal shadows in the posterior muscles. **(D)** High signal was scattered in the tibialis anterior muscle of T2 IDEAL both legs, indicating edema between the muscle tissues.

The patient’s posterior thigh muscles were significantly fatty, the quadriceps and *gracilis* muscles were relatively damaged, a small part of the *tibialis anterior* muscle was retained in both calves, and the posterior muscles were completely fatty (Figure 3).

### Genetic Testing

**Figure 4:**
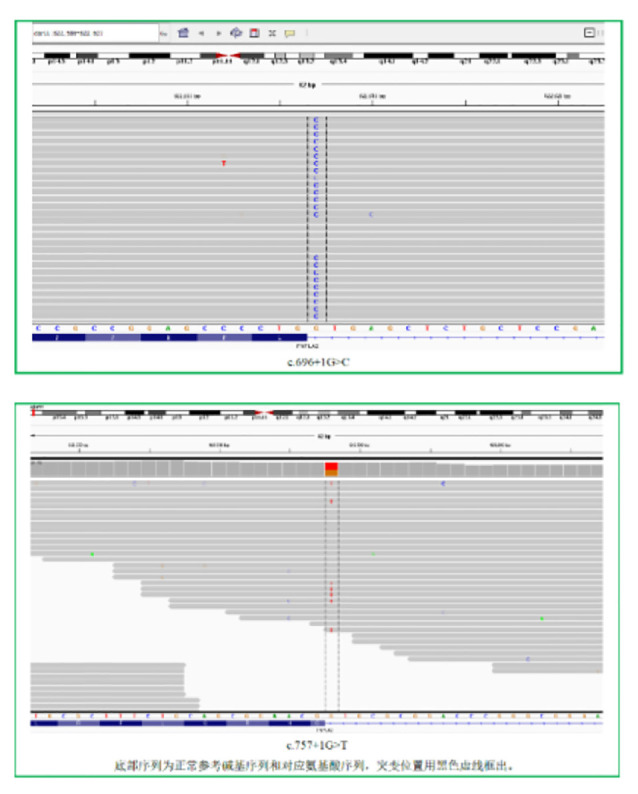
Two heterozygous mutation sites were found in the exon region of the PNPLA2 gene.

Two heterozygosity processes of PNPLA2 gene were detected in exon regions No. 5 and No. 6: c.696 + 1G > C; c.757 + 1G > T (Figure 4).

### Neuroelectrophysiology

Short-term low-amplitude potentials were seen in the examined muscles of the upper and lower limbs, and a small part of the examined muscles showed broad potentials. Sensory motor nerve conduction velocity was normal in the upper and lower limbs.

### Laboratory Examination

CK: 1447 μ/L (25–197 μ/L) CK-MB: 83 μ/L (0–25 μ/L) LDH: 472 μ/L (109–245 μ/L) TC: 4.72 mol/L (2.8–5.2 mol/L), TG: 5.46 mol/L (0.57–1.71 mol/L), LDL: 1.68 mol/L (0–3.1 mol/L).

### Ultrasound

Cardiac ultrasound showed normal atrioventricular size, diminished left ventricular diastolic function, and left ventricular ejection fraction 45%. Abdominal ultrasound: fatty liver.

## Discussion

In 1974, Dorfman (Missaglia et al., 2019) first found lipid droplet deposition in tissue cells of some ichthyosis patients, and then Chanarin et al. (1975) first proposed the diagnosis of neutral lipodeposition (NLSDs) in their study on patients with ichthyosis and myopathy (Elias and Williams, 1985). Fisher et al. (2007) in France reported a case of neutral lipid deposition with mild muscle weakness and no ichthyosis as clinical characteristics and identified the pathogenic gene as *PNPLA2*, which was identified as a subtype of NLSD, namely, neutral lipid storage disease with myopathy (NLSDM).

In normal cells, TGs are stored in the lipid droplets and then hydrolyzed by three different lipases: the lipid droplet-associated adipose triglyceride lipase (ATGL), the cytosolic hormone-sensitive lipase (HSL), and the monoacyl-glycerol lipase. *PNPLA2* codes adipose triglyceride lipase (ATGL), an enzyme that hydrolyzes fatty acids from triacylglycerol. With 504 amino acids, ATGL is the key enzyme in the first step of lipid degradation in muscle and the second incomplete rate-limiting enzyme for non-esterified fatty acid mobilization. It provides a fatty acid moiety that can be used for regulating transcriptional response and mitochondriogenesis. In NLSDM, *PNPLA2* mutations determine partial or total loss of ATGL function causing abnormal accumulation of TGs in many tissues. The increase of TG in the cell can cause and aggravate the accumulation of long-chain fatty acids in the cell, forming a vicious circle and resulting in insufficient cell energy (Hirano et al., 2014). In atrophic and regenerating fibers of patients with NLSD-M, Angelini et al. (2016) and Missaglia et al. (2019) observed TFEB overexpression; in other conditions autophagy markers are increased, suggesting lipophagy has an active role in human lipid metabolism.

Chen et al. (2009) first reported an NLSDM family in China. The authors reported that marginal vacuoles and large fat droplet deposition were observed in muscle fibers of both patients and the intron 2 mutation of the *PNPLA2* gene was detected. Yi et al. (2015) reported the clinical and muscular pathology of three cases of ND with myopathy, but no increase in triglyceride and bordered vacuoles. When our patient first came to our hospital for a muscle pathology examination in 2007, we did not recognize the disease. The muscle pathology report at that time was “Inclusion body myositis with lipid deposition.” In 2015, we performed genetic examination and found two heterozygous mutations in the *PNPLA2* gene, which led to the diagnosis of neutral lipodeposition with myopathy. When the patient came back for a follow-up visit in June 2019, she finished up the relevant examinations of muscle magnetic resonance and peripheral blood Jordans bodies. Together, we had a complete diagnosis from clinical signs and muscle imaging to muscle pathology and genetic testing for this patient.

NLSDM mostly occurs onset in middle age, with an average age of 30 years. It is characterized by symmetric or asymmetric muscle weakness and muscle atrophy. The disease progresses slowly, with heart and liver involvement, but without skin damage (Liang and Nishino, 2011). In this case, the patient started at the age of 33, with the weakness of the proximal lower extremity, followed by the weakness of the right upper extremity, and then the weakness of the left upper extremity, showing asymmetric progression. Unlike patients in previous reports, the patient in this case had proximal limb weakness at the beginning, and distal limb weakness and muscle atrophy gradually appeared as the disease progressed. Eventually, the distal muscle weakness became more severe than the proximal muscle weakness. Although the patient in this case did not have obvious symptoms of heart failure such as palpitations and fug, the color doppler echocardiography indicated that the left ventricular ejection fraction was significantly reduced, indicating that the heart function of the patient had been affected.

The results from muscle biopsy showed significant signs for the diagnosis of NLSDM (Pennisi et al., 2015; Hong et al., 2019). We found two different types of vacuoles in the muscle fibers of this patient. One was a small, evenly distributed sieve-like vacuole caused by fat droplet deposition; the other was made up of rimmed cavities (RV) of varying sizes, the edges of which were caused by the deposition of basophilic particles. This rimmed vacuole is an important pathological change in NLSDM, but RV is not visible in every case (Zhang et al., 2016). In this case, there were a large number of typical RVs in muscle fibers, which is relatively rare. Jordans bodies are another characteristic of NLSDM (Missaglia et al., 2017; Tan et al., 2018). In this case, multiple vacuoles were observed in the cytoplasm of neutrophils by Wright’s staining on peripheral blood smears. “ORO” staining showed that the vacuoles in the cytoplasm were stained as red lipid droplets, showing a typical Jordans corpuscle, which confirmed the lipid deposition in the cytoplasm of neutrophils.

Muscle magnetic resonance imaging (MRI) is the best way to understand the extent of involvement of muscle tissue lesions. In this case, the lower limb muscle magnetic resonance imaging was performed 18 years after the onset of the disease, suggesting that only the *rectus femoris*, medial muscle, *gracilis*, and a small part of the *tibialis anterior* muscle were relatively retained. The thigh muscles, the lateral muscles, the biceps *femoris*, and the calf muscles were completely replaced by fat. Based on current literature, cases with such severe fatty tissues of muscle are rare (Xu et al., 2015; Garibaldi et al., 2017). This may be related to the longer course of the disease from the onset to the diagnosis, which has developed in the late stage of the lesion.

The protein encoded by the *PNPLA2* gene is adipose triglyceride lipase, an important coenzyme in fat metabolism. *PNPLA2* gene mutations include homozygous mutations and composite heterozygous mutations. It has been reported that homozygous mutations are more than composite heterozygous mutations (Pennisi et al., 2017; Zhang et al., 2019). In this patient, two heterozygous mutations were detected in exon regions 5 and 6 of the *PNPLA2* gene, c.696 + 1G > C (guanine > cytosine) and c.757 + 1G > T (guanine > thymine). Both mutation sites are splicing mutations and have been proved to be pathogenic mutations (Lin et al., 2012; Hirano et al., 2014). This mutation affects the activity of adipose triglyceride lipase, leading to the deposition of fat in various tissues and the disease. In this case, serum triglyceride was significantly increased, and Jordans bodies were seen in neutrophils from peripheral blood smears, indicating that lipids are deposited in various tissues in the body. At the same time, studies have shown that the same genetic locus mutations may not have the same clinical manifestations of NLSDM, which may be related to other genes, living environments, and lifestyles of patients (Zhang et al., 2019).

In summary, NLSDM is a clinically rare lipid metabolism abnormal myopathy. The clinical manifestations of patients are very heterogeneous, and the asymmetry of muscle weakness and atrophy are the clinical characteristics. In addition to a large amount of lipid deposition in muscle fibers, some patients may show typical marginal vacuoles. Genetic analysis of *PNPLA2* is necessary to confirm NLSDM diagnosis.

## Data Availability Statement

The raw data supporting the conclusions of this article will be made available by the authors, without undue reservation.

## Ethics Statement

The studies involving human participants were reviewed and approved by the ethics committee of the Jiaozuo People’s Hospital of Henan Province, Henan, China. The patients/participants provided their written informed consent to participate in this study. Written informed consent was obtained from the individual for the publication of any potentially identifiable images or data included in this article.

## Author Contributions

JS was responsible for writing the article. QQ and HLi were responsible for data collection. WC and PC were responsible for making important revisions to the article. YZ was responsible for data analysis. HLv was responsible for providing overall ideas. All authors contributed to the article and approved the submitted version.

## Conflict of Interest

The authors declare that the research was conducted in the absence of any commercial or financial relationships that could be construed as a potential conflict of interest.
